# Biodiversity and Biogeography of Abundant and Rare Microbial Assemblages in the Western Subtropical Pacific Ocean

**DOI:** 10.3389/fmicb.2022.839562

**Published:** 2022-03-30

**Authors:** Qianwen Shao, Dong Sun, Chen Fang, Yunzhi Feng, Chunsheng Wang

**Affiliations:** ^1^Key Laboratory of Marine Ecosystem Dynamics, Second Institute of Oceanography, Ministry of Natural Resources, Hangzhou, China; ^2^Ningbo Institute of Oceanography, Ningbo, China; ^3^College of Oceanography, Hohai University, Nanjing, China; ^4^School of Oceanography, Shanghai Jiao Tong University, Shanghai, China; ^5^Southern Marine Science and Engineering Guangdong Laboratory, Zhuhai, China

**Keywords:** western Pacific Ocean, microbial community, abundant and rare taxa, biodiversity, biogeography

## Abstract

The levels of chlorophyll *a* and nutrient concentrations in the surface waters of the western subtropical Pacific Ocean are among the lowest globally. In addition, our knowledge of basin-scale diversity and biogeography of microbial communities in this vast extremely oligotrophic environment is still rather limited. Here, high-throughput sequencing was used to examine the biodiversity and biogeography of abundant and rare microbial assemblages throughout the water column from the surface to a depth of 3,000 m across a horizontal distance of 1,100 km in the western Pacific Ocean. Microbial alpha diversity in the 200-m layer was higher than at other depths, with Gammaproteobacteria, Alphaproteobacteria, and Clostridia as the dominant classes in all samples. Distinctly vertical distributions within the microbial communities were revealed, with no difference horizontally. Some microbes exhibited depth stratification. For example, the relative abundances of Cyanobacteria and Alphaproteobacteria decreased with depth, while Nitrososphaeria, Actinobacteria, and Gammaproteobacteria increased with depth in the aphotic layers. Furthermore, we found that environmental (selective process) and spatial (neutral process) factors had different effects on abundant and rare taxa. Geographical distance showed little effect on the dispersal of all and abundant taxa, while statistically significant distance–decay relationships were observed among the rare taxa. Temperature and chlorophyll *a* were strongly associated with all, abundant, and rare taxa in the photic layers, while total inorganic nitrogen was recognized as the crucial factor in the aphotic layers. Variance partitioning analysis indicated that environmental selection played a relatively important role in shaping all and abundant taxa, while the variation in rare taxa explained by environmental and spatial processes was relatively low, as more than 70% of the variation remained unexplained. This study provides novel knowledge related to microbial community diversity in the western subtropical Pacific Ocean, and the analyzes biogeographical patterns among abundant and rare taxa.

## Introduction

The western subtropical Pacific Ocean lies within close contact to the Pacific Ocean and the Asian continent, which permits intense land–sea–air interactions, and drives heat and circulation dynamics. Thus, this hydrographically complex area plays a pivotal role in the global climate and environmental change that affects one quarter of the world’s population (Wang, 1999). The chlorophyll *a* and nutrient concentrations of surface waters in the western subtropical Pacific Ocean are among the lowest globally (Longhurst, 2010). Despite the prevalence of oligotrophic characteristics, this region exhibits significant spatial heterogeneity in terms of biological oceanography (Browning et al., 2021), which is mainly constrained by strong and complex western boundary current systems and the warm pool (Barber and Chavez, 1991). Therefore, the western subtropical Pacific Ocean is an ideal area to investigate the relationship between the environment and the biogeography of plankton communities (Rowe et al., 2012).

Microbes play crucial roles in biogeochemical cycling in marine ecosystems (Satinsky et al., 2014) and in the biodegradation of organic compounds (King et al., 2015). By definition, abundant microbial taxa are few in number but high in abundance; they can readily disperse and are thought to provide the most important influxes of dissolved organic matter and carbon cycling in the oceanic ecosystem (Liu et al., 2015). Rare microbial taxa have low abundance and extremely high diversity (Pedrós-Alió, 2012), which contribute to nutrient cycling (Pester et al., 2010); they act as a reservoir that can quickly respond to environmental changes and promote community stability in a wide variety of ecosystems (Shade et al., 2014). The study of both abundant and rare microbial diversity and biogeographical patterns is important. In the open sea, past investigations on abundant and rare microbial taxa have mainly focused on coastal oceans (Campbell et al., 2011; Mo et al., 2018), the Mediterranean Sea (Hugoni et al., 2013; Baltar et al., 2015), and the Arctic Ocean (Galand et al., 2009). The western Pacific Ocean has not received much research attention, so our knowledge of pelagic microbial biodiversity and biogeography in this area is still rather limited (Wang et al., 2020). Recently, Liang et al. (2017) used a flow cytometer to determine the abundances and distributions of picoplankton and virioplankton in the epi-, meso-, and bathypelagic zones of the western Pacific Ocean. With the development of high-throughput sequencing, the diversity and functional structure of pelagic and benthic bacterial assemblages in the western Pacific Ocean were further estimated (Wang et al., 2020), because this technique can help researchers to better identify variation in less abundant species and picoplankton populations when compared with traditional methods. A few studies have analyzed the active planktonic marine archaeal community from the deep chlorophyll maximum (DCM) of the western Pacific Ocean (Dai et al., 2020). Archaea are a major component of the marine ecosystem and play significant roles in the degradation of organic materials (Iverson et al., 2012), which could be 5–10 times less diverse than bacteria (Huber et al., 2007). Overall, for a better understanding of the microbial community in the western Pacific Ocean, there is an urgent need to analyze the abundant and rare taxa separately.

The estimation of the geographic distribution of microbes has been a topic of interest in the scientific community for many years. The knowledge of microbial biogeography directly influences our understanding of their global biodiversity and the underlying processes that shape their distribution. The mechanisms underlying community assembly, which generate the complicated biogeographical patterns of microbe populations, are still central to microbial ecology (Nemergut et al., 2013). Growing evidence demonstrates the importance of two different processes acting to determine the nature of microbial communities. One is a niche-based (environmental selection-related) process (Vellend, 2010) and the other is neutral (dispersal-related) process (Caswell, 1976; Hubbell, 2001). In the western Pacific Ocean, previous studies have shown that environmental factors [e.g., dissolved oxygen (DO), temperature, and salinity] were significantly correlated with microbial community composition and the functional structure of bacterial communities in the epipelagic zone (Wang et al., 2020). Meanwhile, the neutral processes that explain the nature of microbial communities in the western Pacific Ocean have seldom been evaluated. Furthermore, most studies of the biogeographical patterns of distribution in various ecosystems have only investigated the effects of environmental and spatial factors on the composition of the entire microbial community regardless of the distribution of abundant and rare taxa.

The present study used high-throughput sequencing of the 16S rRNA genes to investigate the vertical profiles of microbial communities (including bacteria and archaea) throughout the water column from the surface to the depth of 3,000 m, and their horizontal patterns over a distance of ∼1,100 km in the western subtropical Pacific Ocean, which is a mesoscale survey. Knowledge about the diversity, distribution, and drivers of microbial communities in the western Pacific Ocean, particularly below the photic zone (down 200 m), is only in its nascent phase. Furthermore, we divided microbes into abundant and rare taxa to: (1) compare the biodiversity and biogeographical patterns between them; and (2) quantify the effects of environmental selection and neutral processes on them, which was critical to unveiling the ecological processes and mechanisms involved in maintaining the stability of this ecosystem (Naeem and Li, 1997).

## Materials and Methods

### Sampling Site and Collection

**FIGURE 1:**
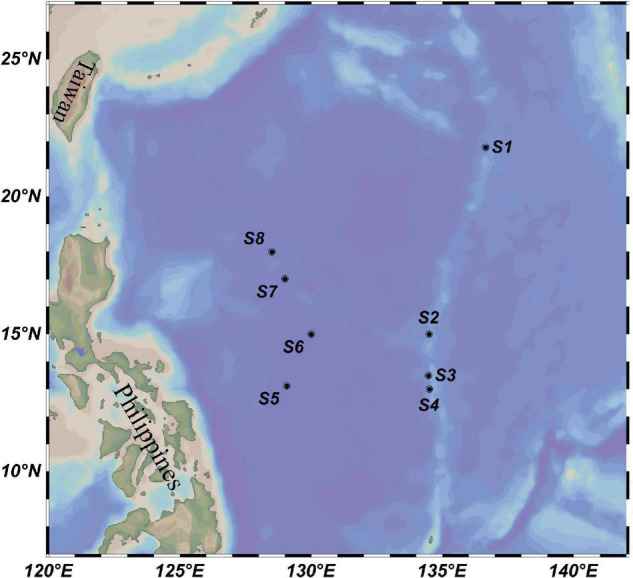
Location of eight water column sampling sites (black circles) in the western subtropical Pacific Ocean.

The investigation was conducted in eight locations in the western subtropical Pacific Ocean, including four sites at the Kyushu–Palau Ridge (S1–S4, 13.00–21.80°N, 134.47–136.64°E) and four sites located in the central deep basin of the Philippine Sea (S5–S8, 13.11–18.00°N, 128.50–130.00°E) (Figure 1). Physical, chemical, and biological investigations were conducted by the research vessel Dayanghao in the above two areas during July and August 2020, as part of the China Ocean Mineral Resources Research and Development Association Cruise DY59. Depths of all stations ranged from 1,629 to 5,648 m, providing us with data on the bathyal environmental to vertically profile the variation of microbial assemblages. A Sea-Bird conductivity-temperature-depth (CTD) sampler (SBE 911 plus, Seabird Co., Ltd., Sunnyvale, CA, United States) was used to collect seawater from six different depths (5 m, 75 m, DCM, 200 m, 500 m, and 3,000 m) at each station. Approximately 4 L of seawater was filtered on board through a 0.22-μm pore-sized membrane (47-mm diameter polycarbonate, Millipore, Bedford, MA, United States). The filters were then frozen at −80°C until further microbial analysis. To prevent contamination between stations, the water filtration system was carefully washed with sterile water before each round of water filtration.

Basic environmental factors of the water column, including depth, temperature, salinity, and DO, were obtained *in situ* using the CTD profiler and a DO sensor during sampling. Other chemical factors, including chlorophyll *a*, dissolved inorganic phosphate, total inorganic nitrogen (including nitrite, nitrate and ammonium) and silicate were measured according to standard methods as previously described (AQSIQ, 2007). The chlorophyll *a* on each filter was measured using a Trilogy Laboratory Fluorometer (Model 7200, Turner Designs Co., Ltd., Sunnyvale, CA, United States) after overnight extraction with 90% aqueous acetone at −20°C in the dark (Parsons et al., 1984).

### DNA Extraction, PCR Amplification, and Illumina Sequencing

Total DNA was extracted from the samples using a DNeasy Power Soil Kit (QIAGEN, Valencia, CA, United States) according to the manufacturer’s instructions. The concentration and purity of the total DNA were measured by a NanoDrop ND–1000 spectrophotometer (Thermo Fisher Scientific, Wilmington, DE, United States). Primers 341F (5′–CCTAYGGGRBGCASCAG–3′) and 806R (5′–GGACTACNNGGGTATCTAAT–3′) were used to amplify the V3 and V4 regions of 16S rRNA (Yu et al., 2005). Adapter sequences were linked to the barcodes at the 5′ end of each primer. The PCR amplification was performed using an ABI 2720 PCR (Applied Biosystems, Foster City, CA, United States) with a total volume of 25 μL containing 5 μL of 5× reaction buffer, 5 μL of 5 × GC buffer, 2 μL of dNTP (2.5 mM), 1 μL of forward primer (10 μM), 1 μL of reverse primer (10 μM), 2 μL of DNA template (20 ng), 8.75 μL of ddH_2_O, and 0.25 μL of Q5 DNA polymerase (0.625 U). The cycling conditions were an initial denaturation at 98°C for 2 min, followed by 25–30 cycles at 98°C for 15 s, 55°C for 30 s, 72°C for 30 s, and a final extension at 72°C for 5 min. Following amplification, the PCR products were purified by Agencourt AMPure XP Beads (Beckman Coulter, Brea, CA, United States), and then quantified using a QuantiFluor^®^ dsDNA System (Promega Corp., Milano, Italy). High-throughput gene sequencing was performed on an Illumina Novaseq-PE250 platform (paired-end reads 2 × 300 bp) by Personal Biotechnology, Co., Ltd. (Shanghai, China).

### Processing of Sequence Data

Raw sequence data were quality filtered and analyzed using QIIME ver. 2.0.0 (Caporaso et al., 2010). Reads were processed by removing tags and primers; the reads with an average quality score <20 and read lengths <150 bp were discarded. After being processed, reads were assembled by FLASH ver. 1.2.7 (Magoč and Salzberg, 2011) with the overlap between R1 and R2 reads >10 bp. High-quality representative sequences for each amplicon sequence variant (ASV) were assigned using UCLUST (Edgar, 2010) with 100% sequence identity. Taxonomic classification was carried out using UCLUST and the Silva 16S rRNA gene database Release 132 (Quast et al., 2013).^1^ The ASVs affiliated with chloroplasts, mitochondria, unclassified groups, and those that were singletons were removed from the dataset. Eventually, to correct possible errors induced by unequal sequencing efforts in all samples, the ASV table was randomly subsampled to ensure an equal number of sequences per sample (53,688) based on MOTHUR v 1.33.3 (Schloss et al., 2009). Next, ASV richness and alpha diversity indices (Pielou’s evenness, Faith’s phylogenetic diversity, Shannon–Wiener and Simpson indices) were calculated from the ASV dataset in QIIME v 2.0.0 (Caporaso et al., 2010). Good’s coverage was calculated in MOTHUR v 1.33.3 software (Schloss et al., 2009).

### Statistical Analyses

All statistical analyses were performed in the R ver. 4.1.1 software.^2^ Prior to statistical analyses, the ASV table were Hellinger-transformed, and the environmental variables were normalized using the “vegan” R package to improve normality and homoscedasticity (Oksanen et al., 2007).

The compositions of microbial communities were visualized at the phylum and class levels using the top 10 in relative abundance and others for both phylum and class. Abundant and rare ASVs were arbitrarily defined based on the ASVs while considering the entire dataset. Abundant ASVs were defined as ASVs with a representation of ≥1% within a sample, and had a mean relative abundance of ≥0.1% in all samples, while rare ASVs were defined as having an abundance <0.01% within a sample, and with a mean relative abundance of <0.001% in all samples. Our definition could avoid any overlap between abundant and rare ASVs when compared with previous studies (Liu et al., 2015). Permutational multivariate analysis of variance (PERMANOVA) was applied to partition the variation in environmental factors and microbial community compositions using the “vegan” (Oksanen et al., 2007) and “ade4” (Dray and Dufour, 2007) R packages. Venn diagrams were used to show the microbial relationships among the six depth groups. In addition, ASV richness and alpha diversity indices were visualized using the “ggplot2” package (Gómez-Rubio, 2017) in R. Wilcox tests were used to evaluate differences between groups using the “ggpubr” package (Kassambara, 2020) in R. Non-metric multidimensional scaling (NMDS) using a Bray–Curtis metric was applied to visualize the overall variation in microbial community composition. A measure of goodness of fit of the ordination was given by a stress value, which was set at <0.20 to minimize misinterpretation (Clarke et al., 2014). An analysis of similarity (ANOSIM) was used to test for significant differences in microbial communities. The global R in ANOSIM ranges from 0 to 1 and represents the degree of separation between groups; *R* = 0 indicates no separation, whereas *R* = 1 suggests complete separation (Clarke and Gorley, 2015). In case of significant differences (*P* < 0.05), the top two ASVs contributing the most to the differences were recognized using similarity percentage analysis (Clarke et al., 2014). The analysis below grouped taxa into three categories: (1) all, (2) abundant, and (3) rare taxa as defined above.

The relationships between the Bray-Curtis dissimilarity of the microbial community and geographic distance and Euclidean distances of environmental factors were analyzed based on Spearman’s rank correlations. The roles of spatial factors were estimated by the procedure coordinates of neighbor matrices (PCNMs, Borcard and Legendre, 2002) method based on the longitude and latitude coordinates of each sampling station. Mantel tests were conducted to investigate the sources of microbial variation while considering both spatial and environmental factors (Diniz-Filho et al., 2013). Redundancy analysis (RDA) was used to explore the effects of spatial/environmental variables on microbial communities based on the longest gradient lengths of detrended correspondence analysis (Oksanen et al., 2007). The longest gradient lengths were <3 for all, abundant, and rare microbial taxa, indicating RDA is suitable. Before the RDA analysis, the environmental factors with high variance inflation factor >50 were eliminated to avoid collinearity among factors, and a forward selection was conducted to select those explanatory factors with significant explaining factors (*P* < 0.05) for further analyses (Blanchet et al., 2008). To test the relative importance of both spatial and environmental factors in structuring microbial communities, we used a variation partitioning approach (Borcard et al., 1992). The analysis decomposed the total variation into fractions that indicate the importance of only environmental variables, only spatial variables, and shared fractions, along with unexplained variation.

## Results

### Environmental Factors of the Water Columns

The environmental factors of each sampling station are shown in Supplementary Figure 1. The highest temperatures (mean ± s.e., 29.90 ± 0.18°C) were observed in surface water and decreased gradually to the lowest depth (1.62 ± 0.02°C) at 3,000 m. Salinity was highest in the DCM (34.95 ± 0.05 psu) and lowest at 5 m (33.99 ± 0.91 psu). Dissolved oxygen increased from 5 m (184.45 ± 15.72 μmol/L) to 75 m (207.20 ± 4.10 μmol/L), and then volatility decreased at 500 m (102.37 ± 30.26 μmol/L) and 3,000 m (138.69 ± 1.19 μmol/L). The concentrations of chlorophyll *a* increased from 5 m (0.06 ± 0.02 μg/L) to the DCM (0.19 ± 0.01 μg/L), then sharply decreased at 200 m (0.05 ± 0.03 μg/L) and were reduced to zero in the deeper layers. The concentrations of nutrients (total inorganic nitrogen, dissolved inorganic phosphate and silicate) continuously increased from 5 m (0.06 ± 0.04 μmol/L, 0.01 ± 0.01 μmol/L, 0.07 ± 0.29 μmol/L, respectively) to 3,000 m depth (39.42 ± 0.01 μmol/L, 2.82 ± 0.02 μmol/L, 146.48 ± 0.01 μmol/L, respectively). Moreover, PERMANOVA tests (Supplementary Table 1) revealed that the six depths significantly differed between each other based on all measured environmental factors (*P* = 0.001), while the eight stations did not differ between each other.

### Overview of Amplicon Sequence Variants and Sequence Numbers

Only 43 DNA samples were studied in detail; three DNA samples (specifically S1–3000, S3–75, S5–DCM) were failed to be collected *in situ*, while the samples of S6–5 and S7–5 were failed to be amplified probably due to improper preservation. In this study, a total of 2,308,584 high-quality microbial V3–V4 reads could be retrieved. Sequence numbers for each sample ranged between 57,468 (S3–5) and 93,579 (S7–200), with an average of 74,072 sequences. Good’s coverage ranged from 99.34% (S7–200) to 99.78% (S3–5) in each sample with the average value of all 43 samples combined at 99.57% (Supplementary Table 2). Based on a clustering threshold of 100%, a total of 37,910 different ASVs were obtained. The total number of ASVs analyzed for each sample varied from 1,188 (S1–DCM) to 2,831 (S3–200), with an average of 1,922 ASVs. Moreover, 78 ASVs (0.21%) with 1,048,240 sequences (45.41%) were considered to be abundant taxa, while 32,235 ASVs (85.03%) with 159,111 sequences (6.89%) were classified as the rare taxa (Table 1). The richness (ASV number) of rare taxa was two orders of magnitude greater than that of abundant taxa, but the abundances (sequence number) of rare ASVs were about one-tenth of the abundant ones.

**TABLE 1:** General description of all, abundant, and rare ASVs datasets at 100% sequence similarity level.

	ASV numbers	Sequence numbers
All ASVs	37910	2308584
Abundant ASVs	78 (0.21%)	1048240 (45.41%)
Rare ASVs	32235 (85.03%)	159111 (6.89%)

### Microbial Community Composition

**FIGURE 2:**
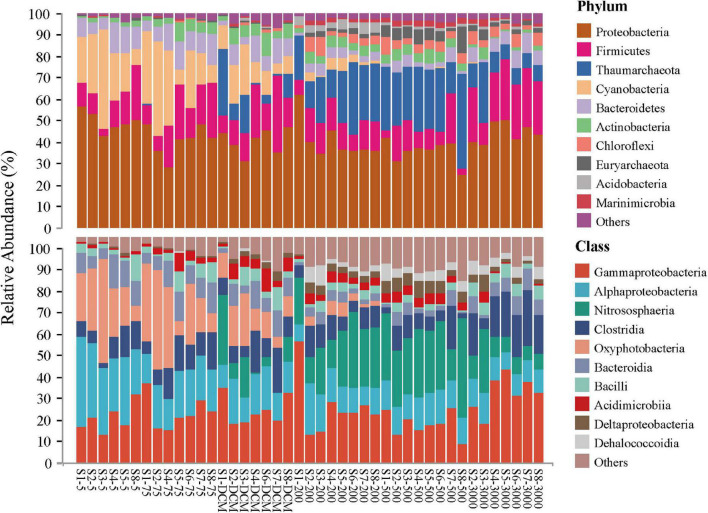
Relative abundance of microbial taxonomic groups at the phylum (top 10 phyla and others) and the class (top 10 classes and others) level across 43 samples.

The top 10 microbial phyla included Proteobacteria (mean relative abundance, 42.02%), Firmicutes (15.37%), Thaumarchaeota (12.89%), Cyanobacteria (9.53%), Bacteroidetes (6.60%), Actinobacteria (3.99%), Chloroflexi (2.51%), Euryarchaeota (2.04%), Acidobacteria (1.13%), and Marinimicrobia (1.12%), which together constituted 97.23% of the total microbial community composition dataset. Generally, the relative contributions of Proteobacteria and Firmicutes were the most abundant phyla at all samples, with the classes Gammaproteobacteria, Alphaproteobacteria, and Clostridia being dominant. Some ASVs belonging to the phyla Cyanobacteria (mainly the class Oxyphotobacteria) and Bacteroidetes (mainly the class Bacteroidia) in the photic layers (5 m, 75 m, and DCM) disappeared or accounted for only a minor proportion of relative abundance in the aphotic layers (200, 500, and 3,000 m). By contrast, the phylum Thaumarchaeota (mainly the class Nitrososphaeria) and Chloroflexi (mainly the class Dehalococcoidia) contributed more to relative abundances in the aphotic layers (Figure 2).

In addition, the most abundant taxonomic groups in both abundant and rare microbial taxa were assigned to Gammaproteobacteria (mean relative abundance: 36.90% ASVs for abundant taxa vs. 15.30% ASVs for rare taxa, respectively) and Alphaproteobacteria (mean relative abundance listed as above: 11.28% ASVs vs. 18.01% ASVs, respectively). Nitrososphaeria and Oxyphotobacteria exhibited higher abundance in the abundant taxa when compared with the rare taxa, while the relative abundance of Clostridia and Bacteroidia were higher in the rare taxa (Supplementary Figure 2).

### Microbial Community Structure Switched Along Depth

The PERMANOVA tests (Supplementary Table 1) showed all, abundant, and rare microbial taxa had significant differences among the six depth groups, and they were not statistically different across the eight stations analyzed here. In terms of the distribution patterns of all ASVs in different layers, 772 ASVs (2.04%) occurred in all depths, while the proportions of unique ASVs were 9.08, 10.28, 12.96, 17.11, 17.15, and 13.65% in the 5-m, 75-m, DCM, 200-m, 500-m, and 3,000-m layers, respectively. All abundant ASVs (100%) were shared among the six depths groups listed above, and 36 ASVs (46.15%) were present at all depths. The percentage of the shared ASVs (10.85%) was much lower for the rare taxa, and only 32 ASVs (0.10%) were found in all depths (Supplementary Figure 3).

**FIGURE 3:**
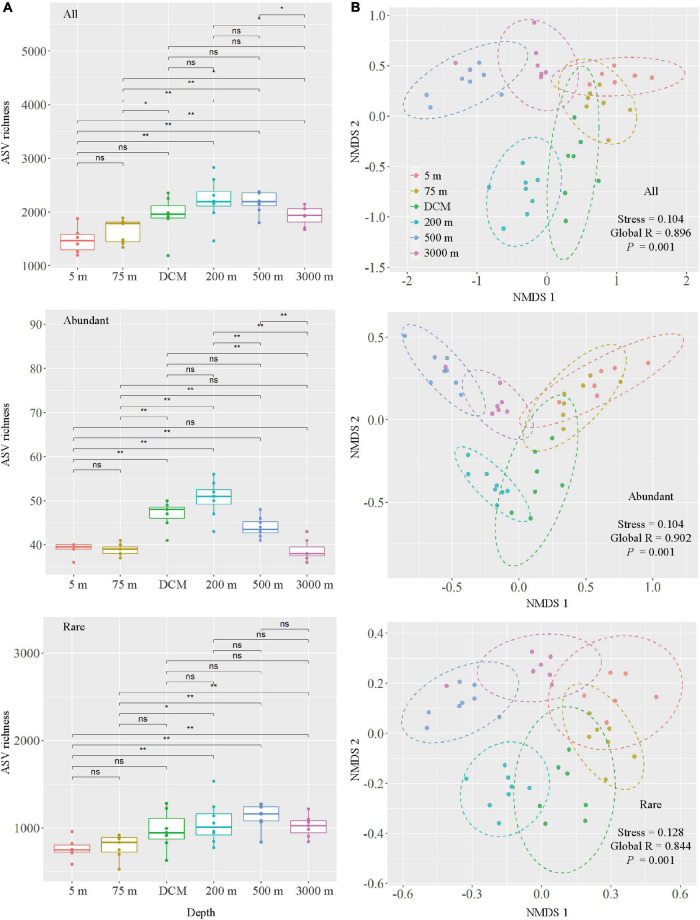
Comparison of microbial communities among six depth groups (5 m, 75 m, DCM, 200 m, 500 m and 3,000 m), DCM, denoted as deep chlorophyll *a* maximum layer. **(A)** Boxplots of ASV richness in six depth groups. Wilcox tests were calculated on the alpha diversity indices between depths. * Represents a statistically significant difference of *P* < 0.05; ** represents a statistically significant difference of *P* < 0.01, ns represents no statistically significant difference. **(B)** Non-metric multidimensional scaling (NMDS) ordination based on Bray–Curtis similarity.

Marked changes in alpha diversity were observed between the different layers of the water column. As expected, the 200-m layer had the highest Pielou’s evenness, Faith’s phylogenetic diversity, Shannon–Wiener and Simpson indices, followed by the DCM layer and the 500-m layer, and the lowest values detected in the 5- and 75-m samples (Supplementary Figure 4). All and abundant taxa ASV richness showed an increasing trend from 5 to 200-m layer, whereas decreasing trend was observed from 200 to 3,000-m layer. For rare taxa, the ASV richness peaked at 500 m, followed by the 200-m layer (Figure 3A). Changes in the ASV richness for all and rare taxa were significant between the 5-m layer and layers deeper than 200 m, as well as between the 75-m layer and layers deeper than 200 m. Meanwhile, changes in abundant taxa ASV richness were significantly different between the 5-m and other layers (except 75 and 3,000 m), 75-m and other deeper layers (except 3,000 m), DCM and 3,000-m layers, 200-m and other deeper layers, and 500 and 3,000-m layers (Figure 3A).

**FIGURE 4:**
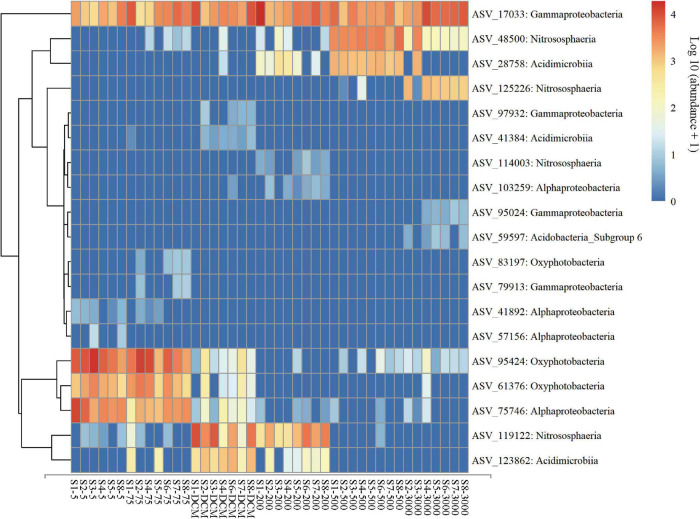
Abundance of the key ASVs identified from similarity percentage analysis for the all, abundant, and rare microbial taxa among six depth groups (5 m, 75 m, DCM, 200 m, 500 m, and 3,000 m). Microbial abundance was scales with log transformation in the heatmap.

The result of NMDS ordination analysis and ANOSIM analyses demonstrated that all (Global *R* = 0.896, *P* = 0.001), abundant (Global *R* = 0.902, *P* = 0.001), and rare (Global *R* = 0.844, *P* = 0.001) microbial taxa were significantly distinct among the six depths groups analyzed here in vertical stratification. All photic samples (5 m, 75 m, and DCM) were separated from the aphotic samples (200, 500, and 3,000 m) by NMDS axis 1 (Figure 3B). Detailed pairwise ANOSIM comparisons of microbial communities also confirmed a significant disparity among different depths (Supplementary Figure 5). Furthermore, rare taxa exhibited striking high values (mean ± s.e., 98.51 ± 0.65%) compared with all (76.85 ± 6.77%) and abundant (51.75 ± 8.93%) taxa, as shown by the pairwise Bray–Curtis dissimilarity of the microbial community among six depth groups (Supplementary Figure 5). The microbial communities in the 200-m water layer appeared to be more dissimilar to those in the 5 m (with higher community dissimilarity for all: 83.86%, abundant: 61.58%, rare: 99.12%) and 75 m (all: 80.87%, abundant: 54.53%, rare: 98.90%) layers than to the other deeper layers (with lower average community dissimilarity for all: 77.38 ± 3.77%, abundant: 46.89 ± 5.43%, rare: 98.41 ± 0.44%) of microbial communities (Supplementary Figure 5). To further explore microbial community dissimilarity, we used similarity percentage analyses to find the qualitative differences causing dissimilarity in all, abundant, and rare microbial taxa among six depth groups. Supplementary Tables 3, 4 shows that 19 ASVs contributed the total differences between two depth groups of samples. Among them, three ASVs belonging to the class Oxyphotobacteria were more abundant in the photic layers, and four ASVs belonging to the class Nitrososphaeria were more abundant in the aphotic layers; other ASVs belonged to the classes Alphaproteobacteria, Acidimicrobiia, Gammaproteobacteria, and Acidobacteria_Subgroup 6 (Figure 4).

### Geographical Patterns of the Microbial Community

**FIGURE 5:**
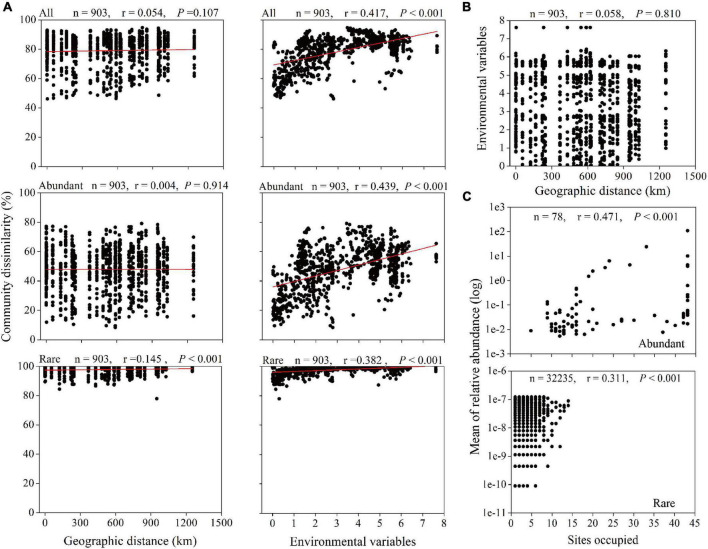
Relationships between microbial community, geographic distance, and environmental factors. **(A)** Spearman’s rank correlations between the Bray–Curtis dissimilarity of microbial community and geographic distance and the Euclidean distance of environmental variables. The *n* is the number of comparisons, and all seven environmental variables were used. **(B)** Spearman’s rank correlation between the Euclidean distance of environmental variables and geographic distance. **(C)** Abundance-occupancy relationship of microbial taxa. Spearman’s rank correlation between mean relative abundance of abundant and rare taxa and number of sites occupied (*n* is the number of ASVs).

Spearman’s correlation between the Bray–Curtis community dissimilarity and geographic distance showed significantly positive correlations for the rare taxa with a correlation coefficient of 0.145 (*P* < 0.001) (Figure 5A). However, the dissimilarity in all and abundant microbial taxonomic compositions exhibited no significant relationship with the geographic distance (*r* = 0.054, *P* = 0.107; *r* = 0.004, *P* = 0.914, respectively). In addition, the Euclidean distance of environmental variables between any two stations or depths did not increase with increasing geographic distance (Figure 5B), but significantly positive correlations were found between environmental variables and all (*r* = 0.417, *P* < 0.001), abundant (*r* = 0.439, *P* < 0.001), and rare (*r* = 0.382, *P* < 0.001) microbial taxa dissimilarities (Figure 5A). In term of the abundance-occupancy relationship, the relative abundance of the abundant and rare taxa and site occupancies were significantly positively correlated (abundant: *r* = 0.471, *P* < 0.001; rare: *r* = 0.311, *P* < 0.001), and no rare ASVs occupied > 35% of samples (Figure 5C).

### Environmental and Spatial Factors Associated With Patterns of Microbial Community

**FIGURE 6:**
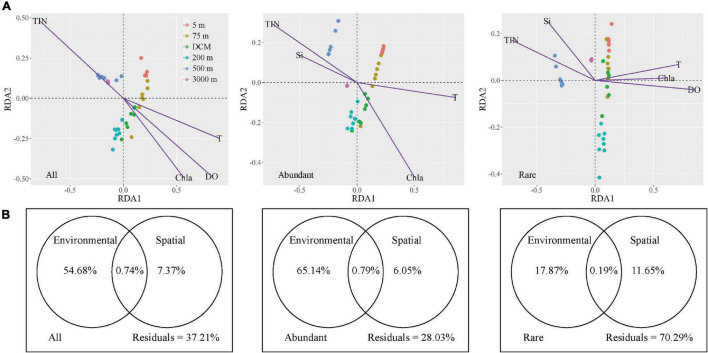
The variation in microbial community explained by spatial and environmental variables, respectively. **(A)** RDA ordinations showing the microbial taxonomic composition in relation to significant (*P* < 0.05) environmental variable. TIN, total inorganic nitrogen, T, temperature, Chla, chlorophyll *a*, Si, silicate, DO, dissolved oxygen. **(B)** Variation partitioning analysis of the microbial taxonomic composition between spatial and environmental variables.

Microbial community assembly, environmental, and spatial factors were analyzed by Mantel tests to explore the effects of environmental (selective processes) and spatial (neutral processes) factors on microbial biogeographical patterns (Supplementary Table 5). Five spatial factors (PCNMs nos. 1–5) exhibited no significant effects on the variation of the all, abundant, and rare microbial taxa, except PCNMs no. 2 was positively correlated with rare taxa (*P* = 0.001). However, seven environmental factors contributed significantly to explaining the community composition of the all, abundant, and rare microbial taxa (*P* < 0.01) (Supplementary Table 5). Redundancy analysis was further used to investigate the relationships between environmental factors and microbial community composition (Figure 6A). All, abundant, and rare taxa in the 5-m, 75-m, and DCM layers were all positively correlated with temperature and chlorophyll *a*, while abundant and rare taxa were all positively correlated with total inorganic nitrogen and silicate in the 500 and 3,000-m layers by forward selection (*P* < 0.05). However, unlike the all and rare taxa, DO did not have significant effects on abundant taxa.

The variation partitioning approach revealed that environmental factors had a stronger influence on microbial community assembly than spatial factors (Figure 6B). For all and abundant taxa, the explained proportion of purely environmental factors (54.68% for all taxa and 65.14% for abundant taxa) tended to be apparently higher than purely spatial factors (7.37% for all taxa and 6.05% for abundant taxa). Shared environmental and spatial factors explained 0.74% of the variation in all microbes, whereas they explained 0.79% community variation of abundant taxa. More importantly, environmental factors (17.87% of pure variance was explained) exhibited a slightly greater influence on rare taxa than spatial selection did (11.65%); this demonstrated that environmental selection and neutral processes explained similar biogeographical patterns of rare taxa. Remarkably, the fact that a large proportion of unexplained variation (70.29%) remained implies that there may be more complex assembly mechanisms that shape the rare microbial assemblages.

## Discussion

### Microbial Community Diversity

Previous studies have indicated that microbial community compositions and genomics on the surface of water columns in the Pacific Ocean vary from those in deeper levels (Delong et al., 2006; Walsh et al., 2016; Wang et al., 2020). Depth is well associated with a dynamic physical and chemical gradient in water columns (Stocker, 2012; Lønborg et al., 2016), which potentially results in a depth-related pattern of microbial distribution (Fuhrman et al., 2015; Guerrero-Feijóo et al., 2017). For example, Walsh et al. (2016) documented that Cyanobacteria, Flavobacteria, and Alphaproteobacteria dominated in the photic layers of the Pacific, whereas the aphotic layers were mainly dominated by Alpha-, Gamma-, Deltaproteobacteria and Deferribacteres. Wang et al. (2020) found Alpha- and Gammaproteobacteria along with Actinobacteria and Cyanobacteria were the dominant phyla in the pelagic western Pacific Ocean; the relative abundance of Cyanobacteria and SAR11 decreased significantly with depth, while that of Actinobacteria and Gammaproteobacteria increased. Our results related to microbial diversity were generally consistent with previous surveys (Figure 2) in that Alpha- and Gammaproteobacteria along with Clostridia were dominant in all samples. The distributions of some bacteria and archaea followed depth stratification; for example, the relative abundances of Cyanobacteria and Alphaproteobacteria decreased with depth, while those of Nitrososphaeria, Actinobacteria, and Gammaproteobacteria increased in the aphotic layers (Figure 4 and Supplementary Table 4). By coupling generation sequencing with statistical analyses, significant vertical differences were detected among six depth groups in all, abundant, and rare microbial taxonomic compositions in our dataset (Figure 3B), rather than being separated by stations in the western subtropical Pacific Ocean (Supplementary Table 1).

*Prochlorococcus* (mainly ASV 95,424 and ASV 61,376, Cyanobacteria) were abundant in the photic layers of the western Pacific Ocean (Figure 4 and Supplementary Table 4), which is similar to previous research (Delong et al., 2006; Liang et al., 2017; Wang et al., 2020). *Prochlorococcus*, which are considered the major primary producers in the ocean, are responsible for a large percentage of the photosynthetic production of oxygen (Kaiser and Benner, 2012). *Prochlorococcus* can adapt to the oligotrophic gyres in the western Pacific Ocean by minimizing their cell and genome sizes and maximizing their surface area to volume ratio, hence minimizing their nutrient requirements and optimizing sites for nutrient transport (Martiny et al., 2009; Berube et al., 2015). Except *Prochlorococcus*, Alphaproteobacteria (mainly ASV 75,746 represented by Rhodospirillales; ASV 41,892 and 103,259 represented by SAR11; and ASV 57,156 represented by SAR116) also dominated in the photic layers (Figure 4 and Supplementary Table 4), which is consistent with the results of Wang et al. (2020). Members of the SAR11 clade represented the most abundant and ubiquitous bacterioplankton in the global ocean (Yooseph et al., 2010), which is suggestive of depth-variable adaptation to light intensity and nutrient availability (Delong et al., 2006). Such adaptations could include their small size, allowing more efficient absorption of nutrients, and their genome streamlining leading to resource specialization (Grote et al., 2012). Rhodospirillales occurred abundantly in the Pacific Ocean (Liu et al., 2019); these taxa have been reported to function as a sulfur-oxidizer (Zhang et al., 2017). The SAR116 clade has been regarded as a ubiquitous but uncultured lineage of marine bacterioplankton (Oh et al., 2010).

Previous studies conducted in deep water masses of the North Atlantic (Sogin et al., 2006), the North Pacific Ocean (Wu et al., 2013), the Greenland Sea (Zaballos et al., 2006), and global seawater ecosystems (Zinger et al., 2011) have also shown Actinobacteria and Gammaproteobacteria had higher representation in the deep-sea microbial community compared to the photic layers. Gammaproteobacteria are known to be highly versatile both morphologically and phylogenetically (Williams et al., 2010), which may explain the colonization of a large range of ecological niches. For example, we found a high relative abundance of Aquabacterium (Figure 4, ASV 17033, Betaproteobacteriales, Gammaproteobacteria) in the entire water column of the western subtropical Pacific Ocean. Bondici et al. (2014) found the nature of Aquabacterium detected in the water column and the presence of reduced iron suggested that complex hydrocarbons were available for their growth and geochemical change, such as iron reduction. These observations raise questions on their adaptation to the marine environments they inhabit and the ecological functions they probably perform. Actinobacteria were found in small proportions in marine environments and almost nothing is known about their role in this habitat (Crump et al., 2004). The Sva0996 marine group (Figure 4, ASV 28758, 123862 and 41384, Microtrichales, Acidimicrobiia, Actinobacteria) has been retrieved occasionally from marine seawaters and sediments (Li et al., 2021). Orsi et al. (2016) found that this group preferred the free-living lifestyle in the seawater and had the ability to assimilate phytoplankton-derived dissolved protein. The adaptive advantage of these microbes may allow them to thrive under extreme low-nutrient conditions of the deep ocean (Wu et al., 2013). Moreover, the relative abundance of archaeal populations (especially for thaumarchaeotal Nitrosopumilales) among all microbes increases gradually with depth (Figure 2), which is consistent with the results of Dai et al. (2020). Previous study has confirmed that the Nitrosopumilales are one of the most abundant and cosmopolitan chemolithoautotrophs (Könneke et al., 2005) and can grow in extremely oligotrophic environments (Schleper and Nicol, 2010). Considering the low substrate concentration in the deep sea, Nitrosopumilales may be responsible for much of the ammonia oxidation that they use for their common metabolism of aerobic ammonia oxidation (Pester et al., 2011).

More interestingly, the highest proportions of unique ASVs (Supplementary Figure 3) and highest alpha diversity indices (Supplementary Figure 4) were found in the 200-m layer, while ASV richness increased from the surface to the mesopelagic zone (Figure 3A, 200 m for all and abundant taxa, 500 m for rare taxa), and decreased with depth. At present, consensus on the generality of trends in microbial alpha-diversity along vertical gradients has not yet been reached in the study area, as well as across the global ocean (Sunagawa et al., 2015). The trend observed in this study is in part consistent with the previous findings observed in the western Pacific Ocean (Wang et al., 2020). The 200-m layer represented a depth of major transitions of temperature, salinity, dissolved oxygen, chlorophyll *a* and major nutrients (Supplementary Figure 1). Sharp chemical/physical gradients might result in rapid speciation (Ghiglione et al., 2012), which prevented a strong dominance by any particular species, and supported a wide range of niches (Schnetzer et al., 2011). Moreover, Supplementary Figure 5 shows the microbial communities in the 200-m layer appeared to be more similar to deep layers communities from DCM to 3,000 m than at the surface and in the 75-m layer, although the 200-m layer was more similar to the photic zone in terms of environmental parameters than to the aphotic layers (Supplementary Figure 1). Similar phenomena occurred in the vertical distribution of the ciliate community in the western Pacific Ocean (Zhao et al., 2017). Therefore, we also argue that the 200-m layer might provide potential species for bathypelagic zones.

### Effects of Environmental Selection and Neutral Processes on Abundant and Rare Microbial Taxa

Previous studies have shown that dispersal limitation caused by geographical distance is one of the important factors that shape the microbial community in the ocean (Sunagawa et al., 2015; Wu et al., 2017). We found that abundant and rare microbial taxa yielded different biogeographical patterns (Figure 5A). Geographic distance seems to have little effect on the dispersal of all and abundant taxa over a spatial scale of 1,100 km in the western subtropical Pacific Ocean, which is consistent with the finding of previous studies in the open sea (Gimmler et al., 2016). The Tara Oceans dataset showed that community dissimilarity had a significantly positive correlation with geographic distance for all organismal size fractions over a transect of ∼6,000 km (De Vargas et al., 2015). Unexpectedly, a statistically significant distance–decay relationship between the rare taxa was observed (Figure 5A), indicating that the distribution of rare taxa in the seawater is somewhat restricted and that these taxa had specific biogeographical distributions. Figure 5C also shows the abundant taxa had higher probability of dispersal than rare taxa according to the site occupancy. Hanson et al. (2012) reported that dispersal may counteract microbial compositional differentiation and weaken the distance–decay relationship. The difference in the distribution patterns of rare and abundant taxa reflected the variation in their ability to disperse and their niche widths. Our observation corroborates the paradigm that the level of dispersal differs in relation to the relative abundance, where more abundant taxa exhibit higher chances of dispersal (Liu et al., 2015; Mo et al., 2018). However, our results were in contrast to Wu et al. (2017), who found that abundant picoeukaryotic communities had greater dispersal limitations when compared with rare taxa in the surface layer of the northwestern Pacific Ocean. The disagreement might be attributed to the difference between microbes and picoeukaryotes.

Except dispersal limitation, microbial geographic patterns are also shaped by environmental heterogeneity (Hanson et al., 2012). In our study, all environmental factors had significant correlations with the all, abundant, and rare microbial taxa (Supplementary Table 5). Figure 6A shows that temperature and chlorophyll *a* were recognized as the crucial factors shaping all, abundant, and rare microbial taxa in the photic layers, while total inorganic nitrogen was found to have a closer relationship with their communities in the aphotic layers. Recent studies focusing on microbes showed that temperature shapes community structures, and various increments in temperature selectively promoted the growth of specific bacterial populations (Lindh et al., 2013). Jiao et al. (2008) reported that both the abundance of aerobic anoxygenic phototrophic bacteria (AAPB) and AAPB relative to total bacteria were positively correlated with the concentration of chlorophyll *a* in the Pacific, Atlantic, and Indian oceans. Inorganic nutrients are essential for the growth and development of microorganisms and different microorganisms can adapt to their optimal growth concentrations of these nutrients (Follows and Dutkiewicz, 2011). However, unlike the all and rare taxa, DO did not have significant effects on abundant taxa (Figure 6A). That is, DO concentrations were observed to strongly affect particle flux and particle transfer efficiency from the euphotic zone to the deep sea since remineralization of organic particles appears to be oxygen dependent (Cram et al., 2018). Some taxonomic lineages are directly affected by oxygen. Similar findings were reported by Liu et al. (2015) and Mo et al. (2018) in that the dynamics of abundant and rare taxa were constrained by different environmental variables. Overall, the abundant and rare microbial taxa were more or less regulated by environmental factors. However, the response of each group to environmental factors was different due to their specific adaptation strategies. Understanding the molecular mechanisms involved in adaptation to varying environmental conditions will require further study.

We further quantified the relative roles of environmental selection and neutral processes that influence the all, abundant, and rare microbial taxonomic compositions in seawater. Figure 6B reveals that environmental selection accounted for more variations than neutral processes in the all taxa (54.68% compared with 7.37% of pure variation) and abundant taxa (65.14% compared with 6.05% of pure variation), indicating that environmental selection played a more important role in shaping them. In regulating the assembly of rare taxa, purely environmental components (17.87%) were slightly more influential contributors than the purely spatial variables (11.65%) in the western subtropical Pacific Ocean. More interestingly, the variation in rare microbial taxa explained by environmental and spatial processes was relatively low, because more than 70% of the variation remained unexplained (Figure 6B). The high proportion of unexplained variation may have been caused by additional biotic and abiotic factors not examined in this study. For instance, upwelling, the movement of currents, and water masses, which play key roles in the distribution of microbes in the open ocean (Fortunato, 2012), were not measured. In addition, the biotic interactions, such as competitive and cooperative relationships between taxa, strongly contribute to their distribution patterns (Lima-Mendez et al., 2015). A few studies have compared the relative influences of selective and neutral processes for the assembly of abundant and rare microbial taxa in different ecosystems (Pedrós-Alió, 2012; Liu et al., 2015; Liao et al., 2017), but no unified conclusions have been reached. Our study further indicated that the relative importance of environmental selection and neutral processes in shaping the communities varies. Environmental selection had stronger effects on abundant taxa, while neutral processes had stronger effects on rare taxa. These data hint at the complex distribution patterns within microbial communities and suggest that future studies are warranted to analyze the abundant and rare microbial assemblages separately.

## Conclusion

The results of this study showed that different levels of biodiversity and biogeography were detected between abundant and rare taxa in the western subtropical Pacific Ocean. Both selective and neutral processes seemed to drive the assembly of abundant and rare taxa, although their relative contributions to the composition of both communities were different. Future research will evaluate the competitive and cooperative relationships between abundant and rare taxa, and predict their functional structure.

## Data Availability Statement

The datasets presented in this study can be found in online repositories. The names of the repository/repositories and accession number(s) can be found below: https://www.ncbi.nlm.nih.gov/, PRJNA791001.

## Author Contributions

QS and DS conceptualized the study and designed the experiment. QS performed the statistical analysis and wrote the manuscript with contributions from DS and CW. DS, CF, and YF participated in the research cruise and collected data. CW approved the final version of the manuscript. All authors contributed to the article and approved the submitted version.

## Conflict of Interest

The authors declare that the research was conducted in the absence of any commercial or financial relationships that could be construed as a potential conflict of interest.

## Publisher’s Note

All claims expressed in this article are solely those of the authors and do not necessarily represent those of their affiliated organizations, or those of the publisher, the editors and the reviewers. Any product that may be evaluated in this article, or claim that may be made by its manufacturer, is not guaranteed or endorsed by the publisher.

## Footnotes

1
http://www.arb-silva.de

2
http://www.r-project.org
