# Supplementary material for: Biodiversity and Biogeography of Abundant and Rare Microbial Assemblages in the Western Subtropical Pacific Ocean
Source: Front Microbiol. 2022 Mar 30;13:839562. doi: 10.3389/fmicb.2022.839562 (PMC9006148; doi:10.3389/fmicb.2022.839562)
Supplement: Supplementary file 1 [file Data_Sheet_1.docx]

Supplementary Material

**
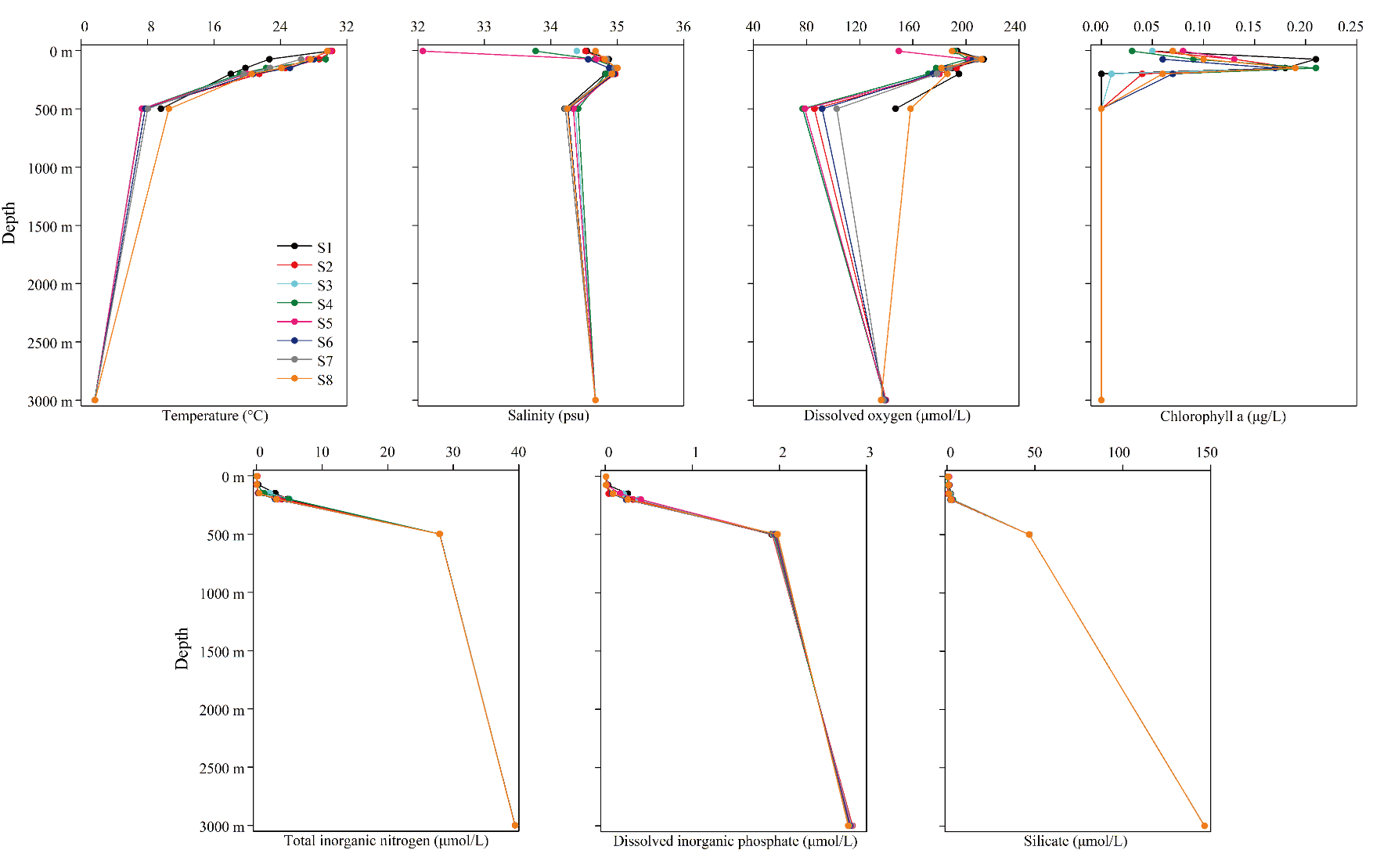
**

**Supplementary Figure 1.** Environmental factors of the water columns at different depths of eight sampling stations in the western subtropical Pacific Ocean.

**
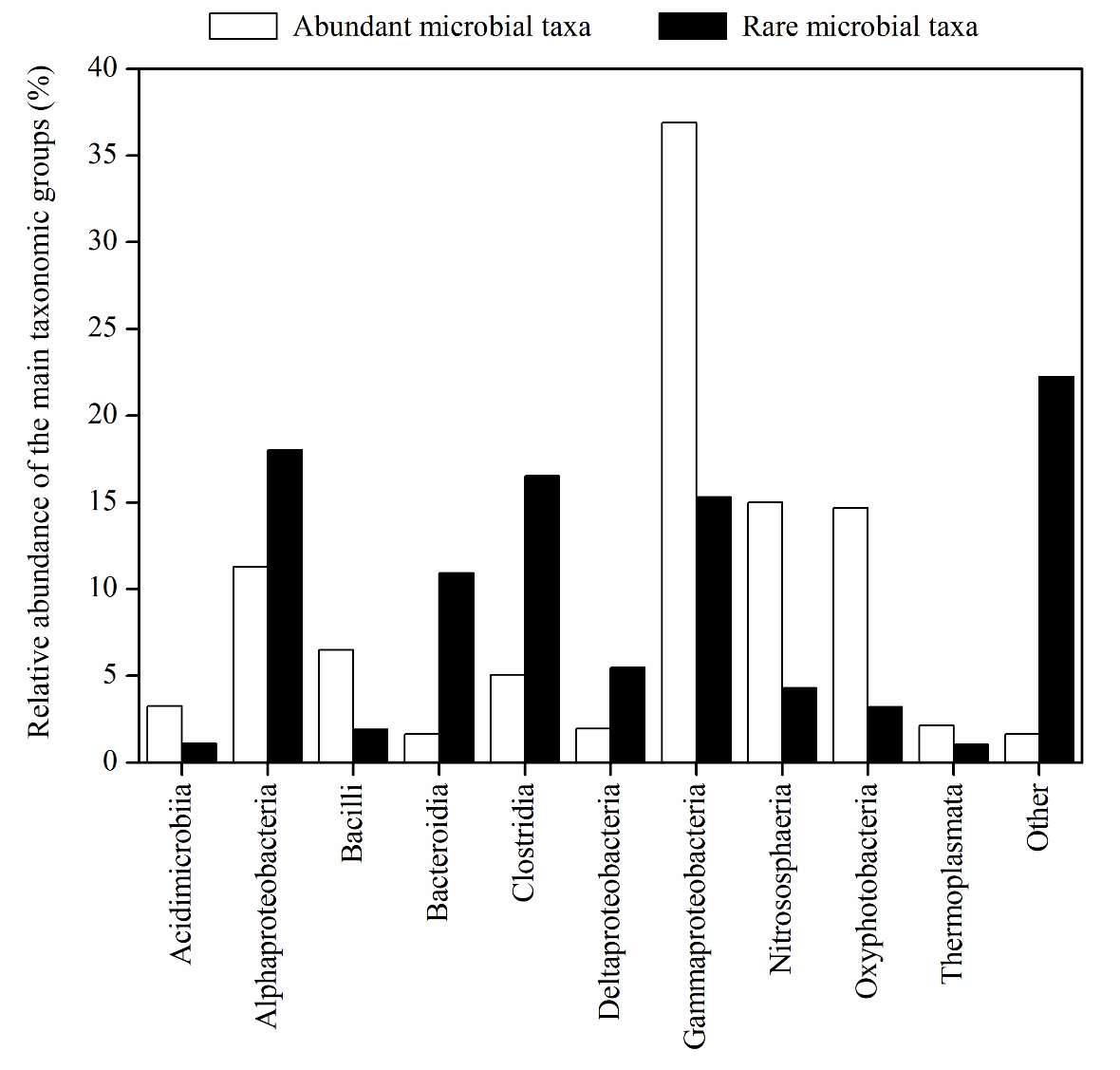
**

**Supplementary Figure 2.** Relative abundance of abundant microbial taxa compared with rare microbial taxa across 43 samples.


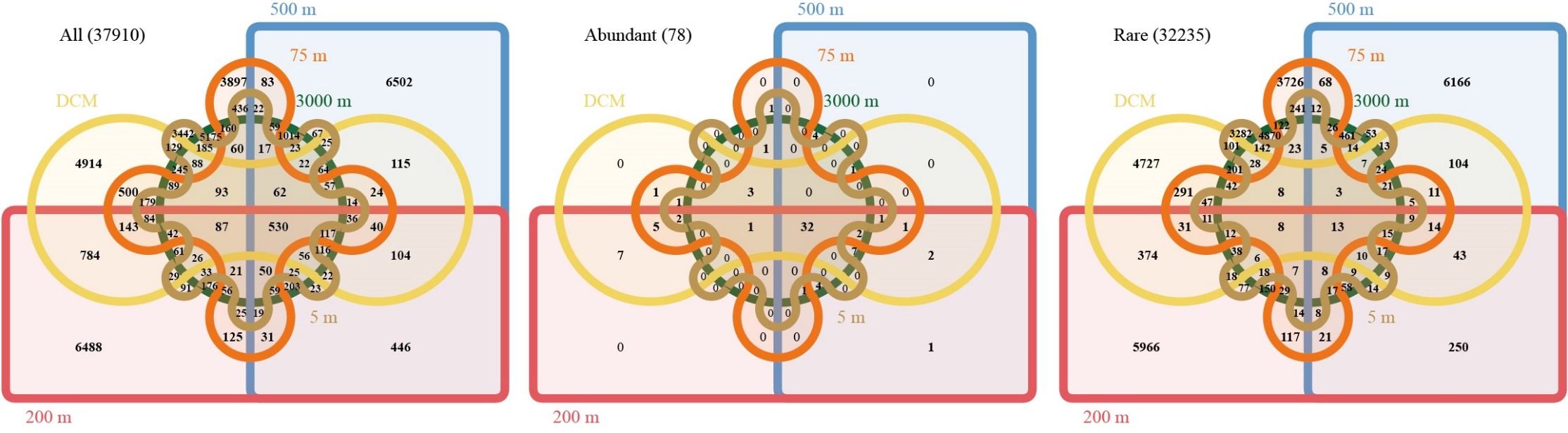


**Supplementary Figure 3.** Venn diagram showing the number of ASVs (37,910 all ASVs, 78 abundant ASVs, 32,235 rare ASVs) that are unique and shared among six depth groups (5 m, 75m, DCM, 200 m, 500 m and 3000 m). DCM, denoted as deep chlorophyll a maximum layer.

**
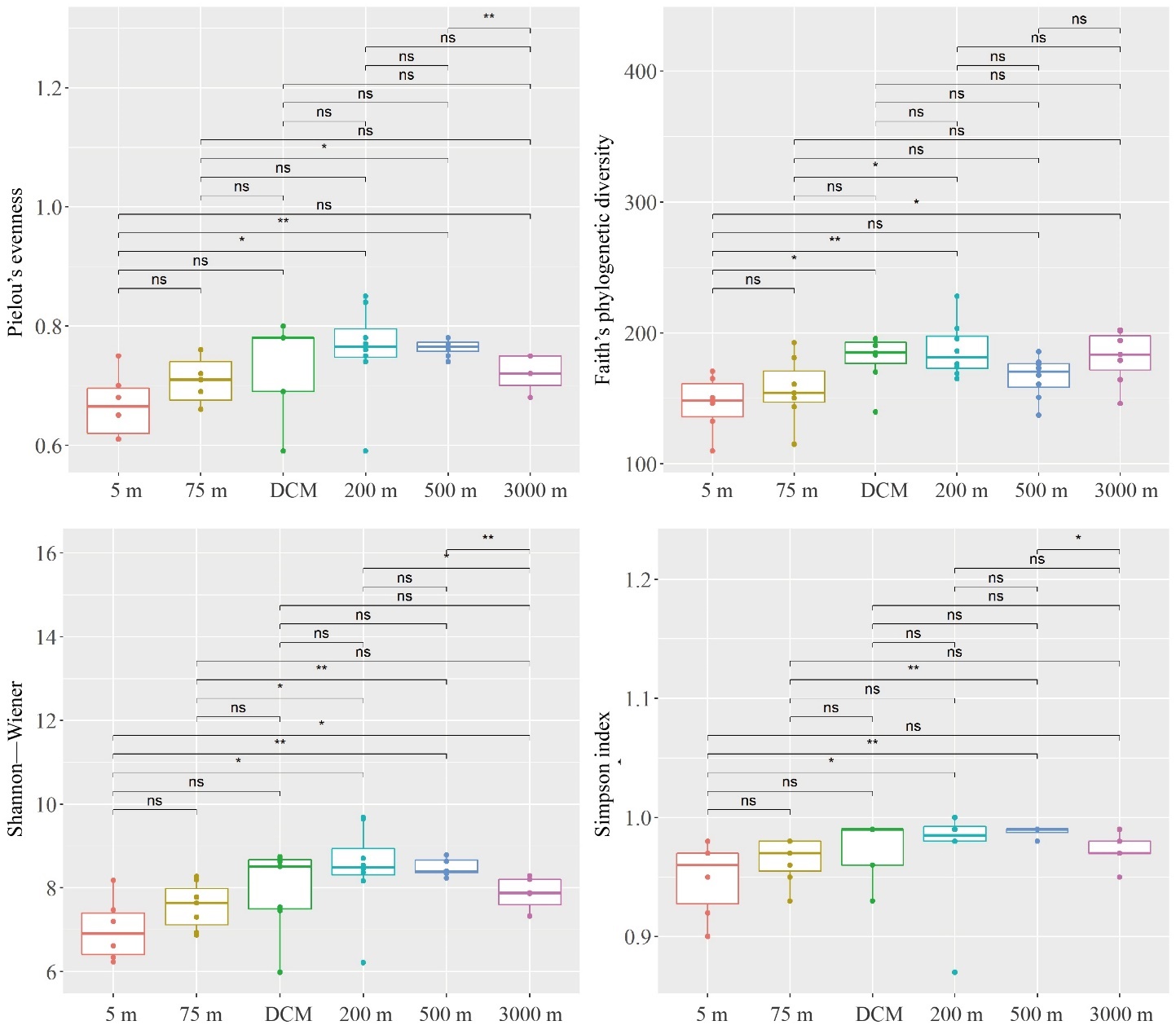
**

**Supplementary Figure 4.** Boxplots of α-diversity indices in six depth groups (5 m, 75m, DCM, 200 m, 500 m and 3000 m), including Pielou’s evenness, Faith’s phylogenetic diversity, Shannon–Wiener and Simpson index. DCM, denoted as deep chlorophyll a maximum layer. Wilcox tests were calculated on the α-diversity indices between depths. * represents a statistically significant difference of *P* < 0.05; ** represents a statistically significant difference of *P* < 0.01, ns represents no statistically significant difference.


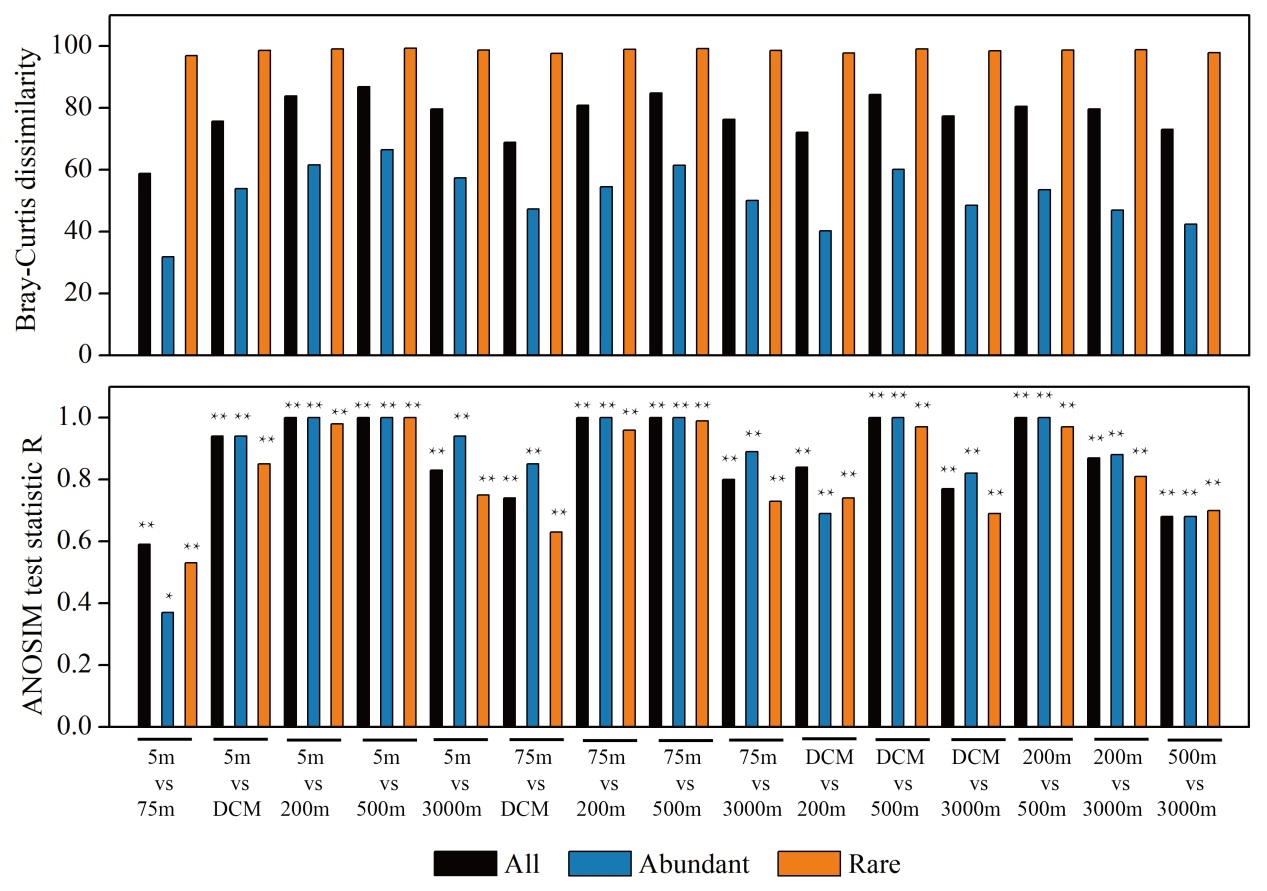


**Supplementary Figure 5.** Analysis of Similarity (ANOSIM) and community dissimilarity (%) for the all, abundant, and rare microbial taxonomic compositions among six depth groups (5 m, 75 m, DCM, 200 m, 500 m and 3000m). DCM, denoted as deep chlorophyll a maximum layer. ANOSIM R- and *P*-values were generated using the Bray-Curtis dissimilarity (*P* < 0.05, denoted as *; *P* < 0.01, denoted as **).

**Supplementary Table 1.** Quantitative effect of different stations and depths on variations in environmental factors and all, abundant, and rare microbial taxa based on permutational multivariate analysis of variance (PERMANOVA).

| Factors | Depths | |  | Stations | |
| --- | --- | --- | --- | --- | --- |
|  | R^2^(Adonis) | *P* |  | R^2^(Adonis) | *P* |
| All environmental factors | 0.888 | 0.001 |  | 0.048 | 0.999 |
| Temperature | 0.984 | 0.001 |  | 0.019 | 0.998 |
| Salinity | 0.480 | 0.001 |  | 0.136 | 0.676 |
| Dissolved oxygen | 0.855 | 0.001 |  | 0.085 | 0.848 |
| Chlorophyll *a* | 0.901 | 0.001 |  | 0.023 | 0.995 |
| Dissolved inorganic phosphate | 0.999 | 0.001 |  | 0.022 | 0.998 |
| Total inorganic nitrogen | 0.999 | 0.001 |  | 0.021 | 0.998 |
| Silicate | 0.999 | 0.001 |  | 0.028 | 0.995 |
| All microbial taxa | 0.554 | 0.001 |  | 0.119 | 0.990 |
| Abundant microbial taxa | 0.732 | 0.001 |  | 0.101 | 0.980 |
| Rare microbial taxa | 0.186 | 0.001 |  | 0.168 | 0.399 |

**Supplementary Table 2.** Summary statistics of sequence counts for each sample before and after quality filtering.

| Samples | Raw reads | High-quality reads | ASV numbers | Good’s coverage (%) |
| --- | --- | --- | --- | --- |
| S1-500 | 93528 | 67457 | 2176 | 99.52 |
| S1-200 | 99133 | 73534 | 1462 | 99.62 |
| S1-DCM | 94849 | 75426 | 1188 | 99.70 |
| S1-75 | 98142 | 66052 | 1417 | 99.67 |
| S1-5 | 89367 | 64361 | 1403 | 99.66 |
| S2-3000 | 96329 | 74599 | 2145 | 99.55 |
| S2-500 | 99492 | 76856 | 2370 | 99.43 |
| S2-200 | 91685 | 65631 | 2606 | 99.49 |
| S2-DCM | 91657 | 69195 | 1956 | 99.60 |
| S2-75 | 97706 | 81393 | 1344 | 99.71 |
| S2-5 | 90775 | 67918 | 1196 | 99.71 |
| S3-3000 | 92771 | 68817 | 1939 | 99.54 |
| S3-500 | 98365 | 74706 | 2148 | 99.41 |
| S3-200 | 95721 | 64799 | 2831 | 99.42 |
| S3-DCM | 94211 | 67469 | 1876 | 99.53 |
| S3-5 | 80627 | 57468 | 1259 | 99.78 |
| S4-3000 | 102152 | 86434 | 1707 | 99.67 |
| S4-500 | 81625 | 66463 | 1800 | 99.70 |
| S4-200 | 87354 | 66215 | 2184 | 99.58 |
| S4-DCM | 94149 | 82684 | 1907 | 99.77 |
| S4-75 | 87669 | 71354 | 1480 | 99.72 |
| S4-5 | 89199 | 69985 | 1603 | 99.68 |
| S5-3000 | 86159 | 65083 | 1670 | 99.71 |
| S5-500 | 86620 | 59102 | 2039 | 99.59 |
| S5-200 | 111723 | 83871 | 2156 | 99.40 |
| S5-75 | 100293 | 77184 | 1886 | 99.53 |
| S5-5 | 100736 | 72318 | 1878 | 99.49 |
| S6-3000 | 99476 | 85210 | 2060 | 99.63 |
| S6-500 | 95178 | 72820 | 2383 | 99.49 |
| S6-200 | 94294 | 76855 | 1987 | 99.59 |
| S6-DCM | 90676 | 68380 | 2355 | 99.55 |
| S6-75 | 89387 | 66699 | 1783 | 99.64 |
| S7-3000 | 95747 | 76060 | 2066 | 99.59 |
| S7-500 | 91502 | 66517 | 2352 | 99.46 |
| S7-200 | 123768 | 93579 | 2310 | 99.34 |
| S7-DCM | 110127 | 81401 | 2252 | 99.34 |
| S7-75 | 113885 | 90570 | 1817 | 99.47 |
| S8-3000 | 106515 | 90541 | 1925 | 99.66 |
| S8-500 | 111674 | 84654 | 2204 | 99.50 |
| S8-200 | 105295 | 79085 | 2200 | 99.44 |
| S8-DCM | 103345 | 82099 | 1983 | 99.49 |
| S8-75 | 94693 | 67996 | 1814 | 99.53 |
| S8-5 | 108320 | 86282 | 1526 | 99.57 |

**Supplementary Table 3.** Similarity percentage analysis for the all, abundant, and rare microbial taxonomic compositions among six depth groups (5 m, 75 m, DCM, 200 m, 500 m and 3000m). DCM, denoted as deep chlorophyll a maximum layer.

| Factor | All | |  | Abundant | |  | Rare | |
| --- | --- | --- | --- | --- | --- | --- | --- | --- |
|  | Species1 | Species2 |  | Species1 | Species2 |  | Species1 | Species2 |
| 5 m versus 75 m | ASV 95424 | ASV 75746 |  | ASV 95424 | ASV 17033 |  | ASV 83197 | ASV 79913 |
| 5 m versus DCM | ASV 95424 | ASV 75746 |  | ASV 95424 | ASV 75746 |  | ASV 57156 | ASV 41892 |
| 5 m versus 200 m | ASV 95424 | ASV 75746 |  | ASV 95424 | ASV 75746 |  | ASV 57156 | ASV 41892 |
| 5 m versus 500 m | ASV 95424 | ASV 75746 |  | ASV 95424 | ASV 48500 |  | ASV 57156 | ASV 41892 |
| 5 m versus 3000 m | ASV 95424 | ASV 75746 |  | ASV 95424 | ASV 75746 |  | ASV 57156 | ASV 41892 |
| 75 m versus DCM | ASV 95424 | ASV 119122 |  | ASV 95424 | ASV 119122 |  | ASV 83197 | ASV 79913 |
| 75 m versus 200 m | ASV 95424 | ASV 61376 |  | ASV 95424 | ASV 61376 |  | ASV 83197 | ASV 79913 |
| 75 m versus 500 m | ASV 95424 | ASV 48500 |  | ASV 95424 | ASV 48500 |  | ASV 83197 | ASV 79913 |
| 75 m versus 3000 m | ASV 95424 | ASV 17033 |  | ASV 95424 | ASV 61376 |  | ASV 83197 | ASV 79913 |
| DCM versus 200 m | ASV 119122 | ASV 17033 |  | ASV 119122 | ASV 17033 |  | ASV 41384 | ASV 114003 |
| DCM versus 500 m | ASV 48500 | ASV 119122 |  | ASV 48500 | ASV 119122 |  | ASV 41384 | ASV 97932 |
| DCM versus 3000 m | ASV 119122 | ASV 125226 |  | ASV 119122 | ASV 123862 |  | ASV 41384 | ASV 95024 |
| 200 m versus 500 m | ASV 48500 | ASV 119122 |  | ASV 48500 | ASV 119122 |  | ASV 103259 | ASV 114003 |
| 200 m versus 3000 m | ASV 119122 | ASV 17033 |  | ASV 119122 | ASV 125226 |  | ASV 103259 | ASV 114003 |
| 500 m versus 3000 m | ASV 48500 | ASV 28758 |  | ASV 48500 | ASV 28758 |  | ASV 95024 | ASV 59597 |

**Supplementary Table 4.** Key ASVs identified from similarity percentage analysis for the all, abundant, and rare microbial taxa.

| Key ASVs | Phylum | Class | Order | Family | Genus |
| --- | --- | --- | --- | --- | --- |
| ASV 61376 | Cyanobacteria | Oxyphotobacteria | Synechococcales | Cyanobiaceae | *Prochlorococcus MIT9313* |
| ASV 95424 | Cyanobacteria | Oxyphotobacteria | Synechococcales | Cyanobiaceae | *Prochlorococcus MIT9313* |
| ASV 83197 | Cyanobacteria | Oxyphotobacteria | Chloroplast | Chloroplast | *Chloroplast* |
| ASV 119122 | Thaumarchaeota | Nitrososphaeria | Nitrosopumilales | Nitrosopumilaceae | *Candidatus Nitrosopelagicus* |
| ASV 48500 | Thaumarchaeota | Nitrososphaeria | Nitrosopumilales | Nitrosopumilaceae | *Nitrosopumilaceae* |
| ASV 125226 | Thaumarchaeota | Nitrososphaeria | Nitrosopumilales | Nitrosopumilaceae | *Nitrosopumilaceae* |
| ASV 114003 | Thaumarchaeota | Nitrososphaeria | Nitrosopumilales | Nitrosopumilaceae | *Nitrosopumilaceae* |
| ASV 75746 | Proteobacteria | Alphaproteobacteria | Rhodospirillales | Aegean169 | *Aegean169* |
| ASV 41892 | Proteobacteria | Alphaproteobacteria | SAR11 | Clade I | *Clade Ib* |
| ASV 57156 | Proteobacteria | Alphaproteobacteria | Puniceispirillales | SAR116 | *Candidatus Puniceispirillum* |
| ASV 103259 | Proteobacteria | Alphaproteobacteria | SAR11 | Clade I | *Clade Ib* |
| ASV 28758 | Actinobacteria | Acidimicrobiia | Microtrichales | Microtrichaceae | *Sva0996* |
| ASV 123862 | Actinobacteria | Acidimicrobiia | Microtrichales | Microtrichaceae | *Sva0996* |
| ASV 41384 | Actinobacteria | Acidimicrobiia | Microtrichales | Microtrichaceae | *Sva0996* |
| ASV 17033 | Proteobacteria | Gammaproteobacteria | Betaproteobacteriales | Burkholderiaceae | *Aquabacterium* |
| ASV 79913 | Proteobacteria | Gammaproteobacteria | Cellvibrionales | Spongiibacteraceae | *BD17* |
| ASV 97932 | Proteobacteria | Gammaproteobacteria | Legionellales | Legionellaceae | *Legionella* |
| ASV 95024 | Proteobacteria | Gammaproteobacteria | EPR3968-O8a-Bc78 | EPR3968-O8a-Bc78 | *EPR3968-O8a-Bc78* |
| ASV 59597 | Acidobacteria | Subgroup 6 | Subgroup 6 | Subgroup 6 | *Subgroup 6* |

**Supplementary Table 5.** Mantel tests for the correlations between spatial / environmental factors (Euclidean distance) and β-diversity of all, abundant, and rare microbial taxa (Bray-Curtis dissimilarity) with 999 permutations.

| Factor | All | |  | | Abundant | | |  | | Rare | | |  |
| --- | --- | --- | --- | --- | --- | --- | --- | --- | --- | --- | --- | --- | --- |
|  | ρ | *P* | |  | | ρ | *P* | |  | | ρ | *P* | |
| All spatial factors | -0.084 | 0.999 | |  | | -0.071 | 0.975 | |  | | -0.022 | 0.725 | |
| PCNM no.1 | -0.032 | 0.865 | |  | | -0.011 | 0.561 | |  | | 0.014 | 0.290 | |
| PCNM no.2 | 0.122 | 0.006 | |  | | 0.017 | 0.326 | |  | | 0.140 | 0.001 | |
| PCNM no.3 | -0.126 | 0.998 | |  | | -0.074 | 0.926 | |  | | -0.094 | 0.994 | |
| PCNM no.4 | -0.141 | 0.999 | |  | | -0.103 | 0.987 | |  | | -0.126 | 0.999 | |
| PCNM no.5 | 0.003 | 0.450 | |  | | 0.024 | 0.343 | |  | | 0.033 | 0.185 | |
| All environmental factors | 0.529 | 0.001 | |  | | 0.485 | 0.001 | |  | | 0.442 | 0.001 | |
| Temperature | 0.592 | 0.001 | |  | | 0.574 | 0.001 | |  | | 0.469 | 0.001 | |
| Salinity | 0.122 | 0.008 | |  | | 0.220 | 0.003 | |  | | 0.095 | 0.002 | |
| Dissolved oxygen | 0.429 | 0.001 | |  | | 0.393 | 0.001 | |  | | 0.319 | 0.001 | |
| Chlorophyll *a* | 0.333 | 0.001 | |  | | 0.288 | 0.001 | |  | | 0.334 | 0.001 | |
| Dissolved inorganic phosphate | 0.517 | 0.001 | |  | | 0.445 | 0.001 | |  | | 0.404 | 0.001 | |
| Total inorganic nitrogen | 0.518 | 0.001 | |  | | 0.445 | 0.001 | |  | | 0.402 | 0.001 | |
| Silicate | 0.325 | 0.001 | |  | | 0.211 | 0.002 | |  | | 0.280 | 0.001 | |
